# Workload in antenatal care before and after implementation of an electronic decision support system: an observed time-motion study of healthcare providers in Nepal

**DOI:** 10.1186/s12911-025-02868-1

**Published:** 2025-02-18

**Authors:** Emma Radovich, Seema Das, Sulata Karki, Christian Bottomley, Ona L. McCarthy, Abha Shrestha, Loveday Penn-Kekana, Rajani Shakya, Biraj Man Karmacharya, Abha Shrestha, Oona M. R. Campbell, Giorgia Gon

**Affiliations:** 1 Faculty of Epidemiology and Population Health, London School of Hygiene & Tropical Medicine, London, UK; 2 Research and Development Division, Dhulikhel Hospital Kathmandu University Hospital, Dhulikhel, Nepal; 3 Department of Community Medicine, Kathmandu University School of Medical Sciences, Dhulikhel, Nepal; 4 Department of Obstetrics and Gynecology, Kathmandu University School of Medical Sciences, Dhulikhel, Nepal

**Keywords:** Electronic decision support system, Antenatal care, Nepal, Time-and-motion study, Workload, Auxiliary nurse midwives

## Abstract

**Background:**

Healthcare interventions are shaped by the resources needed to implement them, including staff time. This study, part of a process evaluation, aims to compare time spent on antenatal care (ANC) and related recordkeeping in two rural primary-level health facilities in Nepal, before and after implementation of an electronic decision support system intervention to improve ANC quality that required additional electronic documentation.

**Methods:**

The study is a before-and-after, observational time-motion assessment. Researchers used the WOMBAT (Work Observation Method By Activity Timing) software to observe and record activities performed by auxiliary nurse midwives providing ANC in two rounds of data collection. We summed the observation time (in minutes) spent on activity categories for each day of observation, in each round of data collection. For each auxiliary nurse midwife, we estimated the proportion of total observation time spent on activities and compared these proportions before and after intervention implementation. We also compared the mean minutes per day spent on ANC and recordkeeping in the two rounds.

**Results:**

Six auxiliary nurse midwives were observed over two data collection rounds (41 total observation days). Prior to intervention, providers spent 7% of their workday on ANC and 6% on related recordkeeping, and time spent on these activities did not change after intervention implementation. Only one of the six auxiliary nurse midwives demonstrated a statistically significant increase in time spent on ANC and recordkeeping after implementation. There was considerable day-to-day variation in ANC time, and substantial periods of “non-work” time (on break or not engaged in work-related activity). Non-work time reduced from 42% in the first round to 26% in the second round of data collection.

**Conclusions:**

Time spent on ANC and related recordkeeping was low and did not change after implementation of the electronic decision support system. ANC and recordkeeping time was sensitive to day-to-day fluctuations in numbers of women attending for ANC at these rural facilities, which may have masked the intervention’s effects. However, the large amount of non-work time observed suggests time constraints during the workday were not a major factor inhibiting use of the electronic decision support system.

**Supplementary Information:**

The online version contains supplementary material available at 10.1186/s12911-025-02868-1.

## Background

Antenatal care (ANC) provides a platform for health promotion, disease prevention, screening, and treatment of pregnancy-related complications. The World Health Organization (WHO) essential package for ANC emphasises the importance of person-centred, quality care during each ANC visit in order to address the large burden of pregnancy-related mortality and morbidity in low- and middle-income countries (LMICs) [1, 2]. The Mobile health Integrated Rural Antenatal care (mIRA) project sought to test the effectiveness of a tablet-based electronic decision support system (EDSS) on improving the quality of ANC in primary-level health facilities in Nepal and India. EDSS use electronic health records to integrate clinical and demographic data with diagnostic and treatment algorithms that provide prompts to improve guideline adherence [3]. In Nepal, where our study took place, a before-and-after study of the custom-built mIRA EDSS [4] and the WHO digital ANC module [5] sought to evaluate the impact of an EDSS intervention on quality of ANC, and to compare the implementation process of the two EDSS designs [6].

Interventions in healthcare environments are constrained by the resources needed to implement them, including staff time. Information and communication technology interventions, including EDSS, often result in changes to work practices in health facilities, enhancing or disrupting existing patterns of work and communication between healthcare providers and patients [7–10]. In pilot projects, EDSS may be implemented alongside paper-based recordkeeping systems [11], as was the case for the mIRA project. The additional tablet-based recordkeeping requirement takes up staff time. Further, efforts to improve guideline adherence and ensure high-quality ANC visits can result in providers spending more time on clinical care. It is important to assess changes to workload and their implications for clinical care and recordkeeping to understand an EDSS intervention’s intended and unintended effects.

Time-motion studies are increasingly applied to yield important insights on workload and the effects of digital health interventions in resource-constrained settings [8, 11–17]. In its most basic form, a time-motion study consists of an independent observer recording the time it takes for a worker to perform a task and the movements related to it and can offer less biased accounts of how healthcare providers use their time, particularly compared to self-report [18]. Time-motion studies in LMICs have been used to examine the effects of electronic documentation and quality improvement interventions in maternal health [11, 19, 20]. Studies in Ghana, Tanzania and West Bank, Palestine of time spent on ANC after EDSS implementation found no change in direct ANC clinical care but improved time efficiencies in recordkeeping where electronic documentation replaced paper-based records [11, 20].

In Nepal, primary care maternity services are largely provided by auxiliary nurse midwives (ANMs) in rural settings [21]. ANMs are engaged in a wide range of services; a substantial focus of their role is on provision of ANC. The mIRA project trained ANMs in participating facilities in using the tablet-based EDSS to assist them in providing high-quality ANC. Because ANMs are increasingly involved in such a range of services [22–25], little is known about how ANMs spend their time, including how much of their workday is spent on ANC and related activities.

This study, conducted as part of the mIRA project process evaluation in Nepal, aims to compare change in time spent on ANC, and on recordkeeping related to ANC, before and after EDSS intervention implementation. To our knowledge, this is the first time-motion study conducted in Nepal.

## Methods

The study is a two-phase (before and after), observational time-motion assessment focusing on major work activities performed by ANMs who provide ANC.

### Study setting

The mIRA project evaluating the mIRA EDSS and WHO EDSS took place in four districts of Bagmati Province, Nepal at 19 primary-level facilities providing ANC in rural areas [6]. One ANM from each facility attended a three-day workshop at Dhulikhel Hospital in March 2022 to receive training in using their allocated EDSS. It was intended that the trained ANM teach other ANMs at the facility to use the EDSS; all ANMs were eligible to use the EDSS during ANC consultations. Following the training, ANMs received one month of in-facility support in using the EDSS by a trained fieldworker, with full EDSS implementation beginning in May 2022.

As part of the mIRA project’s process evaluation, four facilities were purposively selected for qualitative longitudinal case studies consisting of repeat, unstructured observations and in-depth interviews [6]. The time-motion study was conducted in two Primary Health Care Centers with relatively higher ANC caseloads taking part in these longitudinal case studies, one each implementing the mIRA EDSS and WHO EDSS. ANC coverage is high in Nepal; between 2020–2022 nearly 87% of pregnant women in rural areas of Bagmati Province had at least one ANC visit and nearly 79% had four or more ANC visits [26]. Despite high ANC attendance, the participating facilities were in sparsely populated areas serving relatively few pregnant women. Facility-A, implementing the mIRA EDSS, recorded 749 first ANC visits in the year before the study; facility-B, implementing the WHO EDSS, recorded 224 first ANC visits in the year before the study.

### Study design, tool development and definitions

The study continuously observed healthcare providers throughout their workdays in two rounds of data collection, conducted before and after EDSS implementation. Each round of data collection was conducted over two weeks, 10–12 days of observation per round. Due to the remote locations of the two facilities, the researchers stayed in nearby villages during data collection. Researchers conducted observation sessions during normal facility open hours when ANC is provided: approximately 10:00 to 16:00, Sunday through Friday (360 min per day). Both facilities offered 24 h services for the birthing centre and for emergencies.

All staff providing ANC were eligible for observation; participants included ANMs and one staff nurse with similar duties as an ANM. To the extent possible, the same ANM was observed by the same data collector in both rounds of observation, to control for unmeasured factors that might affect activity recording. In the second round of data collection, we prioritised observing the ANM who had attended the EDSS training workshop. Due to the intense concentration required during data collection, researchers took 5–10 min breaks after every 60–90 min of observation (“observation session”) and took at least one 45 min midday break to eat and rest. Researchers tried to time their breaks alongside those of facility staff and when there were no pregnant women presenting for ANC. Facility-A had a designated break time from 13:00–14:00 each day; facility-B did not have a set break time.

Unlike other time-motion studies of digital health interventions in ANC [11, 20], this study did not base the unit of observation on ANC consultations. This is because of evidence from formative research that ANMs in Nepal work in teams and frequently perform ANC consultations with multiple pregnant women simultaneously, switching between patients when, for example, a patient is sent to the laboratory for blood or urine testing, and resuming the ANC consultation when the patient returns with the test results. Further, there was evidence that some ANC-related recordkeeping occurred after ANC consultations ended [27]. In this study, the unit of analysis was minutes of the ANM’s workday.

As the study focus was on ANC and ANC-related recordkeeping, priority was given to observing the ANM(s) providing ANC on any given day of observation. The researcher would follow the ANM until the end of the workday or until the ANM was no longer available (attending home visits, for example). If an observation ended before the end of the workday, the researcher would switch to observing a different ANM, again prioritising any ANM involved in ANC provision, or if no ANC was being provided, then the ANM who was engaged in work activities, rather than non-work activity. If an observation ended after 15:00 (with less than an hour before facility closing), the researcher would end data collection for the day.

The study used the WOMBAT (Work Observation Method By Activity Timing) software, which enables automatic time stamps, and the recording of multitasking and interruptions [7]. To develop the activity categories, two researchers took notes during preliminary unstructured observations in Dhulikhel Hospital’s obstetrics ward and in a Health Post in Kathmandu district during ANC days to develop the initial framework of actions performed by healthcare providers during ANC consultations. We used the notes, and additionally drew on categorisations used in a time-motion study in ANC in Ghana and Tanzania [11], to group actions into mutually exclusive activity categories with defined scope in order to improve reliability and consistency in data collection (Table 1).

**Table 1:** Activity categories, associated sub-tasks and definitions

Activity category	Sub-tasks	Description of activities
**ANC**	Registration	Issuing the ANC registration number, providing ANC booklet, asking and documenting basic personal and contact information of the client into her ANC booklet and register
	Education and counselling	Educating the pregnant woman on topics such as: danger signs in pregnancy, counselling on diet/nutrition, hygiene, need for immunization, STI prevention, family planning, breastfeeding and birth preparedness. Responding to the pregnant woman’s questions about pregnancy or her care (for example when the woman should return for her next ANC visit)
	History taking	History taking- documenting past obstetric history, medical/surgical history, family history of illnesses, calculating EDD/gestational age. Asking about current symptoms or complaints related to the pregnancy
	Physical examination	General examination from head to toe, handwashing, pallor, looking for signs of oedema and abdominal scars, and the examination of breast and pelvis. Palpation of abdomen, measuring of fundal height, listening to foetal heart sound, checking foetal position presentation, performing ultrasound scan
	Investigation/referral	Advising client to attend for lab test, reviewing lab reports, discussing lab results with the client, referring client to another facility for lab tests; taking of blood samples; testing for haemoglobin, testing blood samples for syphilis and HIV using rapid diagnostic test kits; checking blood grouping of client. Testing of urine for protein, glucose, etc. using dipsticks, USG referral, reviewing USG report
	Drug administration	Dispensing medications or supplements (such as deworming, iron or folic acid tablets); writing prescriptions; administering vaccinations to the ANC client, including tetanus diphtheria (Td); counselling on drug side effects
	Vital signs	Taking and documenting height, weight, blood pressure, pulse, and temperature of the ANC client
	Supervising/delegating/training	Supervising nursing or any paramedical students, training those students during their posting
	Navigating EDSS	Reading or scrolling through tablet
**Recordkeeping**	Client handheld ANC card	Entry into client handheld ANC card
	Paper ANC register	Entry into paper-based ANC register
	Other paper records	Entry into any other paper-based registers (e.g. family planning register, immunization register/card, tally sheet for monthly reporting etc.)
	Other electronic records	Computer data entry (including filling out HMIS)
	EDSS tablet	Entry into tablet (mIRA or WHO EDSS)
**Communication**	Client: chit-chat	Talking with clients about the weather, family, the pandemic, etc
	Client: other	Talking with clients about other issues, not directly related to clinical care
	Colleague: work-related	Talking with colleagues or attending to phone calls about client-related matters
	Colleague: chit-chat	Talking with colleagues about non-work issues
**Family planning**		Provision of family planning commodities, counselling on family planning methods, checking equipment available for family planning (for example autoclaved/sterilized materials for IUCD implant), referring client for family planning services elsewhere, supervising/delegating/training students
**Immunization**		Administering vaccinations to children or non-pregnant adults; preparing immunization equipment and materials. Does not include immunizations given to pregnant women
**Admin**		Meeting with facility staff (including supervisor or colleagues), cleaning facility/equipment, preparation of examination room
**Non-work**		Waiting for any reason, resting, meal or tea breaks, socializing (while not simultaneously doing another activity), attending to non-office/personal phone call or a phone call where subject/reason is not clear
**Out of sight**		ANC or other work, movement or other activity done elsewhere (out of sight of observation)
**Other client services**		Newborn health checks, abortion and related actions, conducting deliveries, postnatal care, outpatient services for non-pregnant clients (such as treatment of minor ailments). This includes record maintenance. Maintaining records of safe abortion services, file documentation and compilation, arranging incentive documents, mid-arm circumference measurement of baby, making delivery file, USG of non-ANC client, vaginal examination of non-ANC clients, delivery, post-natal care, dealing medical abortion cases, COVID-19 vaccination, blood pressure measurement of general patient, handling and supporting emergency cases

### Data quality

SK and SD conducted the observations, one of whom is clinically trained. Prior to the first round of data collection, the two researchers piloted the tool in simultaneous observation sessions over two days at Dhulikhel Hospital’s ANC clinic, observing the same healthcare provider, to check inter-observer agreement. Activity coding and sequencing (including the allocation of the primary activity and secondary activity(ies) when multitasking) were reviewed by a third researcher (GG) and minor adjustments to coding suggested. The researchers conducted a second, three-day pilot in a Primary Health Care Center in Lalitpur district to further check inter-observer agreement. Piloting was considered complete when activity categories were coded reliably and only minor discrepancies of < 1 min in time stamp duration remained.

During data collection, the research team met regularly and communicated via a WhatsApp group. Following observations in each facility, in each round, the team debriefed to ensure consistent approaches and understandings of activity categories. The research team also discussed and reflected on findings not captured in the data entry tool to refine analysis plans and interpretation.

### Data analysis

Analyses were conducted at the level of the ANM as the EDSS was hypothesised to be used differently by each ANM and have differing effects on time use [6]. For each ANM, we summed the total amount of time (in minutes) of observation spent on the activity categories for each day of observation, in each round of data collection. We calculated the proportion of time spent on each activity out of the total amount of observation time for each round. For ANMs observed in both rounds, we used unpaired t-tests to compare differences in daily proportions of time spent on ANC, ANC-related recordkeeping and non-work activity between rounds 1 and 2 (before and after EDSS implementation). For the activities of ANC and ANC-related recordkeeping, we described the total amount of time (in minutes) spent on specific sub-tasks of the activity (Table 1). To compare daily time spent on ANC and ANC-related recordkeeping before and after EDSS implementation, we calculated the mean number of minutes per day spent on ANC and recordkeeping for each ANM, among observation days that included that activity, and used unpaired t-tests to compare differences. We also calculated paired t-tests to compare overall differences for all ANMs combined, between rounds 1 and 2, in proportion of time spent on ANC, ANC-related recordkeeping and non-work activity. Statistical significance was considered at the 5% level. All analyses were conducted in Stata/SE V.16 (StataCorp, College Station, Texas, USA).

The WOMBAT software enabled the documentation of multitasking (doing two or more activities concurrently). We calculated the proportion of total observation time spent multitasking in each round of data collection. For activities with multitasking sequences, time calculations were based on the primary activity to avoid overlapping activity time totals exceeding total observation time. We considered the primary activity to be the first activity in a multitasking sequence or the activity that overlapped with all the simultaneous activities in the multitasking sequence. For example, a five-minute episode of recordkeeping that included one minute of simultaneous communication with colleagues that began 10 s before recordkeeping and was immediately followed by one minute of ANC history-taking, would be coded as a primary activity of recordkeeping. Multitasking sequences could include a simultaneous activity that extended beyond the time period of the primary activity.

Across the two rounds of data collection three time-stamped episodes (between 3 and 41 s in length) were missing an activity code and were excluded from the analysis. Due to a data collection software issue in round 2, ANM-6 in facility-B had duplicate observation sessions documented for one observation day. While the observation session activity codes and time stamps were similar—suggesting consistency in coding—they did not match exactly. As it was not possible to determine which of the duplicated observation sessions were more accurate, one observation day for ANM-6 in round 2 was excluded from the analysis. In round 1, ANM-1 at facility-A was observed for less than five minutes and in round 2, ANM-7 at facility-B was observed for less than 13 min before both ANMs left the facility to attend off-site trainings for the day. These two observation days, for these two ANMs, were excluded from the analysis.

The data collection software allowed the observer to end an activity independently before the start of the next activity (where this was not coded as multitasking). As the software logged continuously elapsing time, this resulted in a time gap, with some observation time not coded to an activity. Less than 1% of observation time (0.0–1.2% per ANM) was not coded to an activity; we retained all observation time (coded to an activity or not) in our denominators.

## Results

Round 1 data collection was completed in December 2021 in facility-A and in February–March 2022 in facility-B. During round 1, we conducted 11 days of observation in facility-A and nine days of observation in facility-B (Table 2). During round 1, facility-B was closed for three days for public holidays. Round 2 data collection was completed in July 2022 in facility-A and in August 2022 in facility-B. During round 2, we conducted 10 days of observation in facility-A and 11 days of observation in facility-B.

**Table 2:** Total amount of observation time and mean observation time per day of observation by facility and ANM in each round of data collection

**A.** Total amount of observation time per facility and average amount of observation time per day in rounds one and two											
		Baseline (round 1)					Endline (round 2)				
		Number of observation days	Total amount of observation time (in minutes)	Mean amount of observation time per day (in minutes)	SD^a^	Range (min–max)	Number of observation days	Total amount of observation time (in minutes)	Mean amount of observation time per day (in minutes)	SD	Range (min–max)
Facility-A		11	5475	497	47	(434–562)	10	4949	501	54	(372–553)
Facility-B		9	4915	547	63	(448–679)	11	5200	500	99	(229–623)
TOTAL		20	10,389	520	60	(434–679)	21	10,149	501	77	(229–623)
**B.** Total amount of observation time per ANM and average amount of observation time per day in rounds one and two											
		Baseline (round 1)					Endline (round 2)				
		Number of observation days	Total amount of observation time (in minutes)	Mean amount of observation time per day (in minutes)	SD	Range (min–max)	Number of observation days	Total amount of observation time (in minutes)	Mean amount of observation time per day (in minutes)	SD	Range (min–max)
Facility-A	ANM-1	9	2136	237	41	(152–294)	0	-	-	-	-
	ANM-2^b^	8	1710	214	73	(92–286)	5	1348	270	26	(226–290)
	ANM-3	7	1629	233	50	(132–283)	8	1938	242	35	(159–265)
	ANM-8	0	-	-	-	-	5	1132	226	40	(167–271)
	ANM-9	0	-	-	-	-	2	531	266	2	(264–268)
Facility-B	ANM-4	5	1429	286	13	(263–297)	4	1206	302	19	(275–321)
	ANM-5^b^	4	1033	258	83	(159–351)	5	1385	277	21	(242–292)
	ANM-6	4	971	243	58	(159–289)	5	1168	234	93	(75–302)
	ANM-7	6	1482	247	69	(121–328)	6	1441	240	63	(114–286)

^a^*SD* standard deviation

^b^ANM who received training in using the EDSS

In facility-A, three ANMs were observed in round 1 (ANM-1, ANM-2 and ANM-3); however, one ANM (ANM-1) was unavailable during round 2 so two new ANMs (ANM-8 and ANM-9), who were not observed in round 1, were observed in round 2. In facility-B, the same four ANMs were observed in rounds 1 and 2 (ANM-4, ANM-5, ANM-6 and ANM-7). Observations were conducted over 2–9 days for each ANM; the six ANMs in both rounds were observed for 4–8 days each (Table 2). The length of observation time of ANMs ranged from 92 to 351 min per day, with a mean amount of observation time per day of 242 min (standard deviation [SD]: 49) in round 1 and 253 min (SD: 49) in round 2. The two EDSS-trained ANMs were ANM-2 in facility-A and ANM-5 in facility-B; both had multiple years’ experience at their facilities and permanent contracts (Supplementary file 1, Table S1).

Time spent multitasking was 3.8% and 3.7% of total observation time in rounds 1 and 2, respectively.

**Fig. 1:**
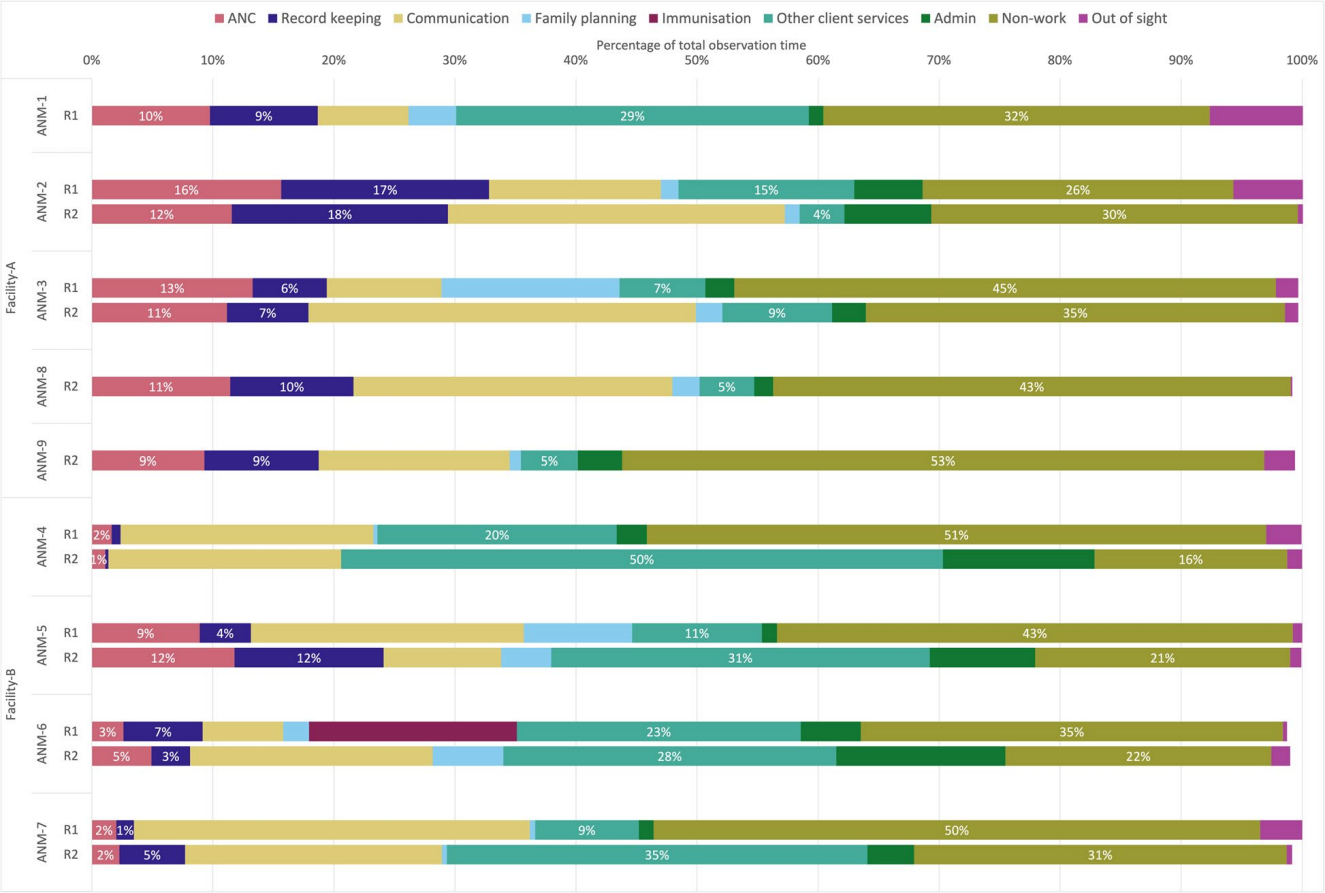
Proportion of total observation time spent on activity by ANM in rounds 1 and 2

Among all the ANMs observed, the proportion of observed time spent on different activities is shown in Fig. 1. Across both rounds, between 1.1–15.6% of observed time was spent on ANC, and 0.3–17.9% of time was spent on ANC-related recordkeeping. In facility-A (mIRA EDSS), the five observed ANMs spent at least 9% of observed time on ANC across both rounds, whereas the three of the four ANMs in facility-B (WHO EDSS) spent less than 5% of observed time on ANC. Across all ANMs, the overall proportion of time spent on ANC (7.4% in r1 vs 7.1% in r2, *p* = 0.849) and on recordkeeping (6.0% in r1 vs 7.6% in r2, *p* = 0.372) was similar in the two rounds. Among the six ANMs observed in both rounds, there was no evidence of change in the daily proportions of time spent on ANC between the two rounds for five ANMs and evidence of an increase for ANM-7 in facility-B (2.6% vs 9.5%, *p* = 0.018, Table 3). There was no evidence of change in the daily proportions of time spent on ANC-related recordkeeping for five of the six ANMs and evidence of an increase for ANM-7 in facility-B (1.8% vs 9.4%, *p* = 0.042, Table 3).

**Table 3:** Comparison of the overall and daily proportions of ANC, recordkeeping and non-work activity time among ANMs observed in rounds 1 and 2

			**ANC**			**Recordkeeping**			**Non-work**		
			A. Proportion of total observation time	B. Mean daily proportion of observation time	*p*-value (column B, r1 vs r2)	A. Proportion of total observation time	B. Mean daily proportion of observation time	*p*-value (column B, r1 vs r2)	A. Proportion of total observation time	B. Mean daily proportion of observation time	*p*-value (column B, r1 vs r2)
Facility-A	ANM-2	R1	15.6%	19.5%	0.335	17.18%	17.9%	0.994	25.7%	24.7%	0.407
		R2	11.6%	11.5%		17.86%	17.8%		30.3%	30.1%	
	ANM-3	R1	13.3%	12.3%	0.777	6.13%	6.6%	0.992	44.7%	46.0%	0.041
		R2	11.2%	11.2%		6.73%	6.6%		34.6%	34.4%	
Facility-B	ANM-4	R1	1.6%	2.8%	0.073	0.74%	1.2%	0.896	51.2%	50.7%	0.100
		R2	1.1%	4.5%		0.26%	1.0%		15.9%	20.7%	
	ANM-5	R1	8.9%	13.0%	0.898	4.24%	4.4%	0.143	42.6%	42.5%	0.035
		R2	11.8%	14.2%		12.33%	14.9%		21.1%	21.1%	
	ANM-6	R1	2.6%	4.5%	0.603	6.54%	8.0%	0.252	34.9%	33.1%	0.666
		R2	4.9%	7.0%		3.18%	3.5%		22.0%	27.1%	
	ANM-7	R1	2.0%	2.6%	0.018	1.45%	1.8%	0.042	50.1%	52.6%	0.064
		R2	2.3%	9.5%		5.43%	9.4%		30.8%	29.5%	

A substantial proportion of observed time was spent on “non-work” activity (which included waiting around, meal or tea breaks, or attending to personal phone calls) (Fig. 1). For all ANMs, approximately a fifth to half of all observed time was coded as non-work across both rounds. On some observation days, the proportion of non-work time constituted the majority of observation time (Supplementary file 1, Figure S1). Overall, there was strong evidence for a reduction in the proportion of non-work time (41.5% in r1 vs 25.8% in r2, *p* = 0.034) before and after EDSS implementation. Table 3 shows that among the ANMs observed in both rounds, there was evidence that daily proportions of time spent on non-work activity decreased for ANM-3 in facility-A (46.0% vs 34.4%, *p* = 0.041) and for ANM-5 in facility-B (42.5% vs 21.1%, *p* = 0.035); marginally significant reductions were also observed for ANM-4 and ANM-7 in facility-B (*p* = 0.100 and *p* = 0.064, respectively).

The aggregate totals concealed large variations in how ANMs’ time was spent day-to-day. The number of minutes spent on ANC varied by observation day, and some observation days included no time spent on ANC (Supplementary file 1, Figure S2). ANMs in facility-A, particularly ANM-2 (the EDSS-trained ANM) and ANM-3, had similarly wide ranges in minutes spent on ANC across the observation days. In facility-B, ANM-5 (the EDSS-trained ANM), had a much wider range in minutes spent on ANC across the observation days compared to their three colleagues. Further, there was variation in time use within the work day, with periods of non-work throughout but particularly in the afternoon (Supplementary file 1, Figure S3).

**Fig. 2:**
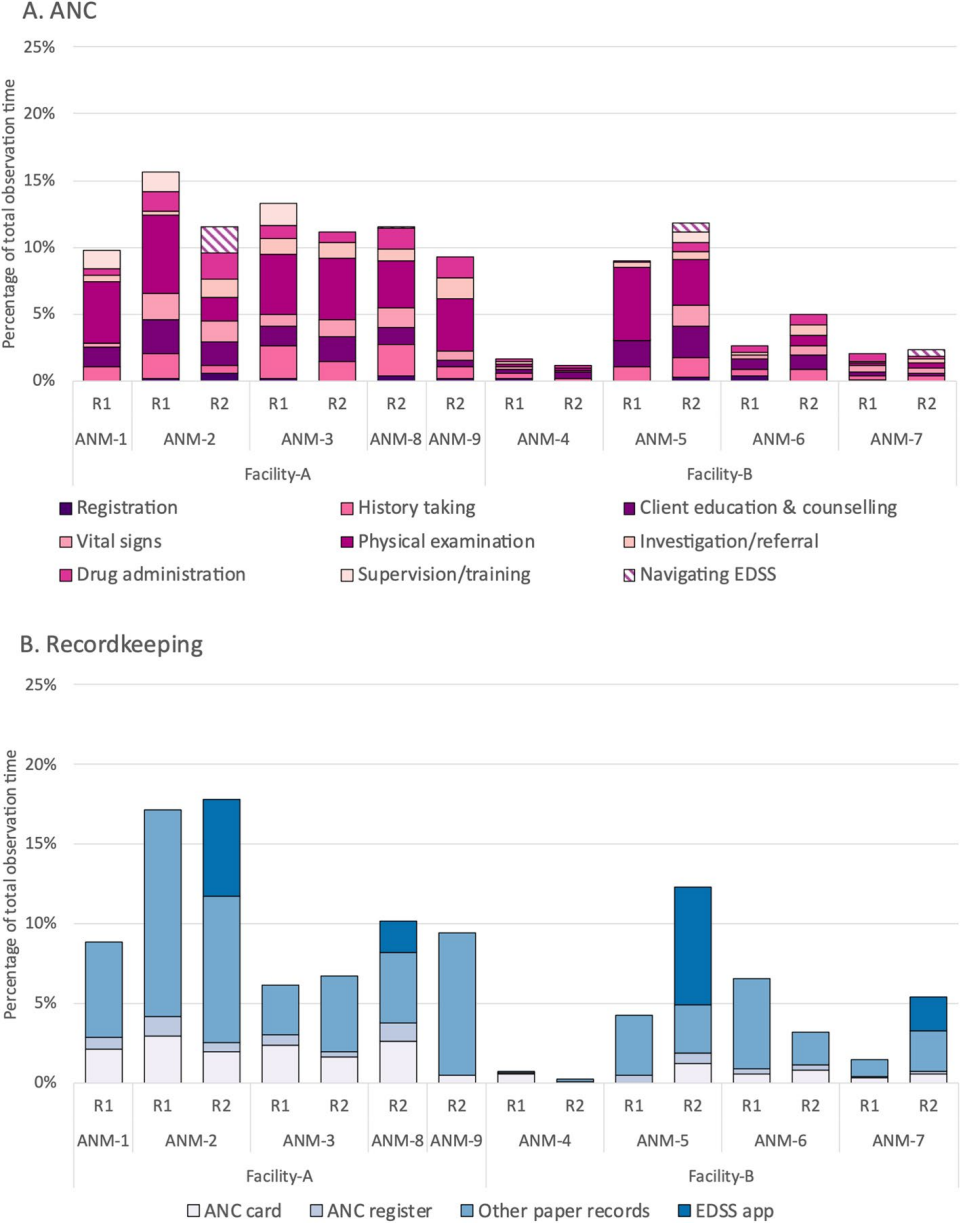
Proportion of total observed time spent on sub-tasks for (**A**) ANC and (**B**) recordkeeping

Fig. 2 shows the activities of ANC and recordkeeping by sub-task. Within ANC, the sub-task of physical examination took up the majority of time spent on ANC for most ANMs. Only three ANMs in round 2 were observed as navigating the EDSS during ANC, including ANM-2 and ANM-5, the two EDSS-trained ANMs (Fig. 2a). Navigating the EDSS (scrolling through the software or reading prompts) accounted for 16.9% and 5.8% of all ANC activity time in round 2 for ANM-2 and ANM-5, respectively. Within recordkeeping, filling in other paper records (which included monthly reporting) accounted for the majority of recordkeeping time for all but ANM-4 in round 1 and ANM-5 in round 2 (Fig. 2b). In round 2, four ANMs were observed recordkeeping in the EDSS app. The two EDSS-trained ANMs, ANM-2 and ANM-5, spent a third to two-thirds of recordkeeping time, respectively, on documentation in the EDSS app.

Table 4 shows the comparison of mean number of minutes per day spent on ANC and recordkeeping in rounds 1 and 2. For all ANMs, mean time spent on ANC and recordkeeping was less than 60 min a day. For ANM-4 and ANM-7, time spent on ANC was less than 20 min per day in rounds 1 and 2. There was no evidence for a change in mean number of minutes per day spent on ANC or on recordkeeping for five of the six ANMs observed in both rounds. For one ANM (ANM-7), time spent on ANC and recordkeeping increased from 6 to 16 min per day for ANC (*p* = 0.029) and from 4 to 20 min per day for recordkeeping (*p* = 0.047) from round 1 to round 2.

**Table 4:** Comparing mean number of minutes per day spent on ANC and recordkeeping in rounds one and two of data collection

**A**. ANC											
		Baseline (Round 1)				Endline (Round 2)				Difference in mean minutes per day, r1 to r2	
		Days of observation that included ANC activities (n)	Mean number of minutes per day	SD	Range (min–max)	Days of observation that included ANC activities (n)	Mean number of minutes per day	SD	Range (min–max)		*p*-value, r1 vs r2
Facility-A	ANM-1	7	30	33	(6–101)	-					-
	ANM-2	7	38	28	(1–77)	5	31	5	(25–36)	-7	0.592
	ANM-3	7	31	23	(1–73)	8	27	15	(5–53)	-4	0.707
	ANM-8	-				5	26	12	(15–44)		-
	ANM-9	-				2	25	2	(23–26)		-
Facility-B	ANM-4	3	8	1	(7–9)	1	14	n/a	n/a	6	0.059
	ANM-5	3	31	26	(6–58)	4	41	37	(3–92)	10	0.705
	ANM-6	2	13	10	(6–20)	3	19	16	(4–36)	6	0.658
	ANM-7	5	6	4	(2–12)	2	16	3	(14–19)	10	0.029
**B**. Recordkeeping											
		Baseline (Round 1)				Endline (Round 2)				Difference in mean minutes per day, r1 vs r2	
		Days of observation that included record keeping activities (n)	Mean number of minutes per day	SD	Range (min–max)	Days of observation that included record keeping activities (n)	Mean number of minutes per day	SD	Range (min–max)		p-value, r1 vs r2
Facility-A	ANM-1	8	24	25	(4–83)	-					-
	ANM-2	7	42	31	(13–102)	5	48	9	(36–60)	6	0.678
	ANM-3	6	17	9	(8–28)	8	16	10	(6–37)	0	0.950
	ANM-8	-				5	23	23	(5–57)		-
	ANM-9	-				2	25	22	(9–41)		-
Facility-B	ANM-4	3	4	4	(0–7)	1	3		n/a	0	0.930
	ANM-5	4	11	7	(4–19)	4	43	35	(2–88)	32	0.128
	ANM-6	3	21	16	(5–37)	4	9	6	(2–15)	-12	0.226
	ANM-7	5	4	4	(1–11)	4	20	14	(0–30)	15	0.047

## Discussion

This time-motion study in Nepal examined changes in time spent on ANC and ANC-related recordkeeping before and after the implementation of a tablet-based EDSS, which was introduced alongside paper-based recordkeeping. We found ANMs in the study facilities spent a small proportion of their workday on ANC and related recordkeeping, and this did not change after implementation of the EDSS. ANC took up no more than 16% of any ANM’s workday in either round, and ANC-related recordkeeping was no more than 18%. There was considerable day-to-day variation in the proportion of time spent on ANC, including days where ANMs were observed doing no ANC activity. Substantial periods of the ANMs’ workdays were spent on “non-work”, or not engaged in any work-related activity or on breaks, and there is evidence that non-work time reduced after EDSS implementation.

Only one of the six ANMs observed in both rounds showed evidence of a change in the proportion of time and mean number of minutes per day spent on ANC and recordkeeping after EDSS implementation. ANM-7 was observed to have a statistically significant increase in mean number of minutes per day spent on both ANC and on recordkeeping, though these activities still accounted for less than 10% of the ANM’s total observation time. Notably, the two ANMs who received training in the EDSS did not have statistically significant changes in mean time per day spent on recordkeeping, though this may reflect the high degree of day-to-day variability in ANC and related recordkeeping activity. However, both EDSS-trained ANMs spent large proportions of their recordkeeping time on entering data in the EDSS in round 2.

The overall reduction in non-work time before and after EDSS implementation should be interpreted cautiously as this may reflect fluctuations in service demand and is unlikely to be related to the intervention. The large amount of non-work time observed suggests that time constraints during the workday as a whole were not a major barrier to infrequent use of the EDSS documented in other studies [27]. However, non-work time should be considered within the context of variable patient flow, for ANC and other services. For instance, in round 1, the four ANMs at facility-B spent between 8.6–23.5% on other client services and in round 2 spent between 27.5–49.7% of observed time on other client services, which included attending to women giving birth. As part of ethnographic work conducted in the study facilities for the mIRA project process evaluation, we observed that the facilities were often relatively busy with patients (including for ANC) in the morning due to local bus schedules and few, if any, patients attended the facilities in the afternoon (Karki S, Das S, Radovich E, Shrestha A, Shakya R, McCarthy OL, et al.: The implementation realities of a digital antenatal care improvement intervention in Nepal: insights from ethnographic work in primary health facilities, in preparation). Further, ANMs often described this non-work time as waiting around for patients to arrive and did not view it as break time. The finding of substantial non-work time is similar to findings from studies in other LMICs, including time-motion studies in India which found ANMs spent considerable amounts of time waiting for patients to arrive on designated clinic days [17, 28]. A study of nurses in reproductive and child health clinics in Tanzania, found variation in staff productivity was largely explained by patient flow but that nurses rarely demonstrated the initiative to undertake other tasks (such as filling in health management information system forms) when patients were not present [13].

ANMs spent very little time on ANC. The low proportion of time spent on ANC may reflect how the workload was managed with multiple ANMs involved in ANC consultations, reducing the total amount of time individual ANMs were observed performing ANC tasks. The distribution of daily time spent on ANC between teams of ANMs at both facilities (see Supplementary file 1, Figure S2) suggested different ways ANC was managed. ANMs in facility-A appeared to work as more of a team to provide ANC, so when the facility was busy, all the ANMs were busy providing ANC. In facility-B, ANM-5 appeared to frequently do much more ANC compared to colleagues, suggesting that on many days ANM-5 was providing most ANC direct patient care. However, even when spread across multiple ANMs, the low mean number of minutes per day spent on ANC suggested that ANMs spent short periods giving direct clinical care to pregnant women—a finding supported by other studies in the mIRA project (Karki S, Das S, Radovich E, Shrestha A, Shakya R, McCarthy OL, et al.: The implementation realities of a digital antenatal care improvement intervention in Nepal: insights from ethnographic work in primary health facilities; in preparation) [29]. The amount of time dedicated to ANC raises questions about whether good quality care can be provided within such short time periods. Our study found no evidence that time spent on ANC changed—though small increases in ANC time were noted for one of the six ANMs—and the overall mIRA project evaluation found no improvement in quality of care after implementation of the EDSS [29]. Our findings on minimal changes in time spent on ANC is echoed in other studies of EDSS interventions in ANC, which found no change in time spent on clinical care in ANC [11, 20], though the study in Palestine found improvements in ANC guideline adherence [30] while the study in Ghana, Tanzania and Burkina Faso saw no evidence for improvement in quality of care [31].

Overall time spent on ANC-related recordkeeping was low, and non-ANC related recordkeeping was captured under other activity categories, such as family planning, immunization or other client services, limiting our ability to parse the full recordkeeping workload of ANMs. Our findings about the low proportion of time spent on recordkeeping should be interpreted carefully due to the design of the data collection tool (and ANC focus of the study) as this stands in contrast to the substantial literature on the burden of documentation in healthcare. Other studies in LMICs have found a much larger recordkeeping burden in primary care facilities [32, 33], including in ANC [34].

### Limitations

The study offers unique insights but was not without limitations. The resource-intensive data collection of the time-motion study design meant spreading observation time over 3–4 ANMs in each around of data collection. The ANM who would be selected to receive EDSS training was unknown during round 1 of data collection, so observation time was distributed across all ANMs, limiting the number of workdays observed for each ANM. Efforts were made to observe the EDSS-trained ANMs in round 2 as much as possible, though these ANMs were sometimes unavailable. The limited days of observation per ANM, alongside the high degree of day-to-day variability, may have masked any potential effects of EDSS implementation on time spent on ANC or related recordkeeping. However, evidence from other studies in the mIRA project suggested that the EDSS was rarely used and did not significantly change ANC practices, supporting the findings of no meaningful change in time use in this study [27, 29].

Due to the intensity of data collection, as well as ANMs going off-site for periods of the day, it was not possible to observe every minute of the workday. This may have underestimated time on some activities. Due to the data collection approach, where observers tried to time their own breaks with that of the ANMs, non-work time in this study may be underestimated. However, priority was given to observing ANMs providing ANC during observation days, and researchers tried to time their breaks when the ANMs were not involved in clinical activities, reducing the likelihood that ANC and related recordkeeping time were underestimated. Further, for all ANMs, most observation days included at least 252 min of observation (70% of normal facility open hours), and more than 80% of observation days included at least 216 min of observation (60% of facility open hours).

Before-and-after studies are limited in accounting for other contextual factors that may have contributed to changes (or lack of change) observed. There was a longer than anticipated gap between baseline data collection and the start of EDSS implementation, resulting in more than six months between rounds 1 and 2, though this was a comparatively shorter baseline to endline gap than other studies. The time-motion study in Ghana and Tanzania was completed six months before EDSS implementation and then 17 months after implementation [11]. However, during the six-month gap in this study, there were several staffing changes that impacted data collection, including ANMs returning from or going on maternity leave or being re-assigned to a newly opened surgical ward. As a result, six ANMs could be observed in both rounds, only two in facility-A. The staffing changes may have impacted the team working and specific tasks of individual ANMs, which we were unable to account for in our study design.

Finally, there is a risk of bias introduced by the Hawthorne effect where ANMs may have changed their behaviour and usual working patterns as they were conscious of being observed. However, others have argued that busy healthcare providers are less able to alter their work patterns even if being observed [16]. We saw no evidence of the participants in our study altering their behaviour to appear more favourable to observers, given the large amount of non-work time.

## Conclusion

We did not find evidence that the EDSS intervention substantially changed the amount of time ANMs spent on ANC or related recordkeeping. ANC and ANC-related recordkeeping constituted a small proportion of ANMs’ time during the workday. Lack of staff time during the workday was unlikely to have been a major factor in the low uptake of the EDSS intervention that was observed in other studies in the mIRA project. However, we found it challenging to conduct a time-motion study in low-volume facilities where time spent on ANC and recordkeeping was sensitive to day-to-day fluctuations in women attending for ANC and demands from other client services. Future time-motion studies would be more feasible in less remote settings serving larger numbers of pregnant women or in facilities with designated ANC clinic days to focus data collection resources on sampling a greater number of observation days with ANC activity to increase the power to detect potential changes resulting from EDSS interventions.

## Supplementary Information

Additional file 1.

Additional file 2.

## Data Availability

The datasets analysed during the current study are available from the corresponding author on reasonable request.
