# Supplementary material for: Workload in antenatal care before and after implementation of an electronic decision support system: an observed time-motion study of healthcare providers in Nepal
Source: BMC Med Inform Decis Mak. 2025 Feb 18;25:87. doi: 10.1186/s12911-025-02868-1 (PMC11834578; doi:10.1186/s12911-025-02868-1)

Supplementary Material

Table S1 *Characteristics of healthcare providers observed in the study*

		Attended EDSS workshop	Contract type	Qualification	Years of work experience (as an ANM or equivalent)	Years employed at facility	Skilled Birth Attendant (SBA) trained
**Facility-A**	ANM-1		permanent	ANM	5	<1	yes
	ANM-2	yes	permanent	ANM	5.5	3	yes
	ANM-3		temporary contract	ANM	8	3	yes
	ANM-8		permanent	ANM	unknown	4	yes
	ANM-9		temporary contract	ANM	10	<1	yes
**Facility-B**	ANM-4		permanent	staff nurse	3	1.5	yes
	ANM-5	yes	permanent	ANM	8	8	yes
	ANM-6		permanent	ANM	8	2	yes
	ANM-7		temporary contract	ANM	7	<1	yes

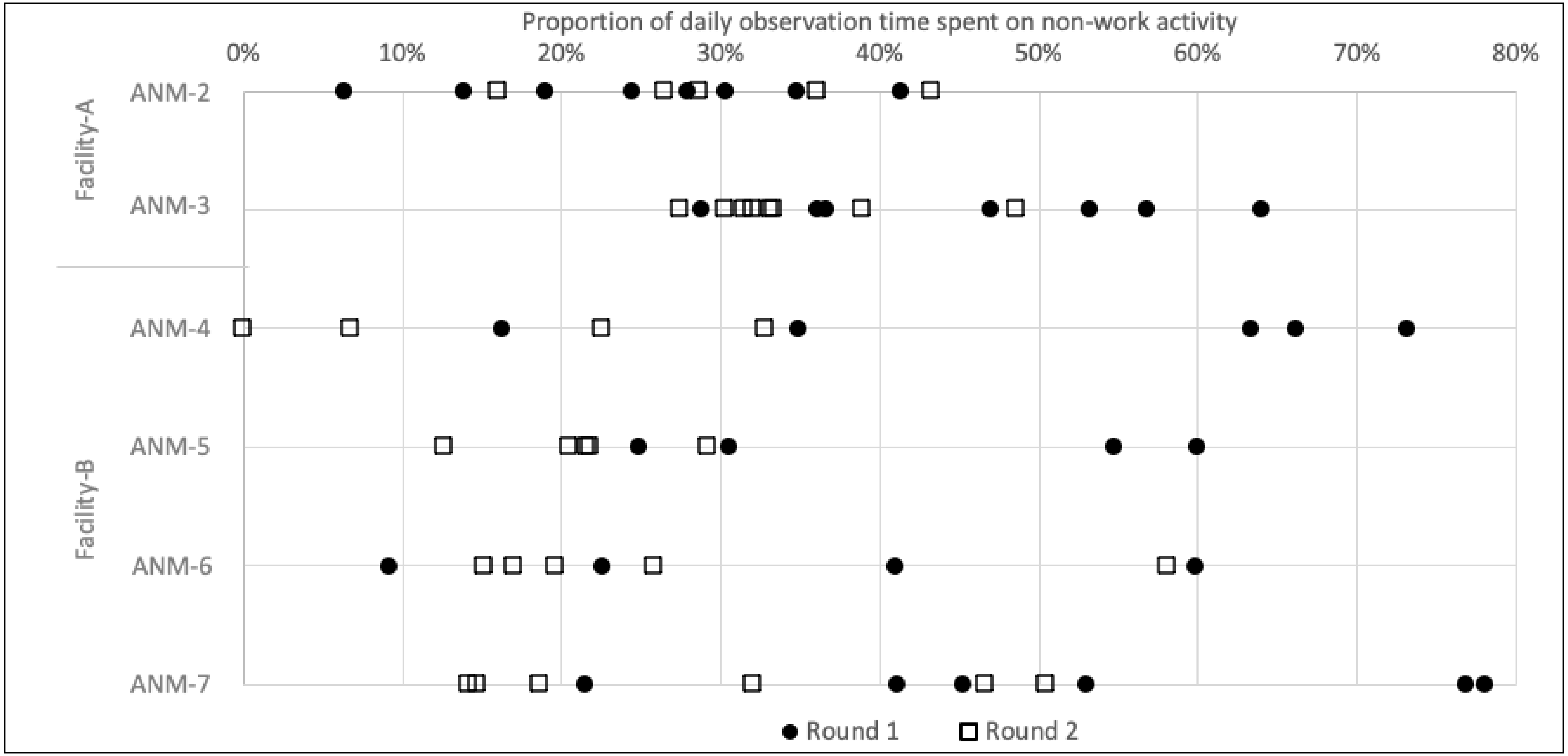

Figure S1 *Daily proportion of observation time spent on non-work among ANMs observed in rounds 1 and 2*

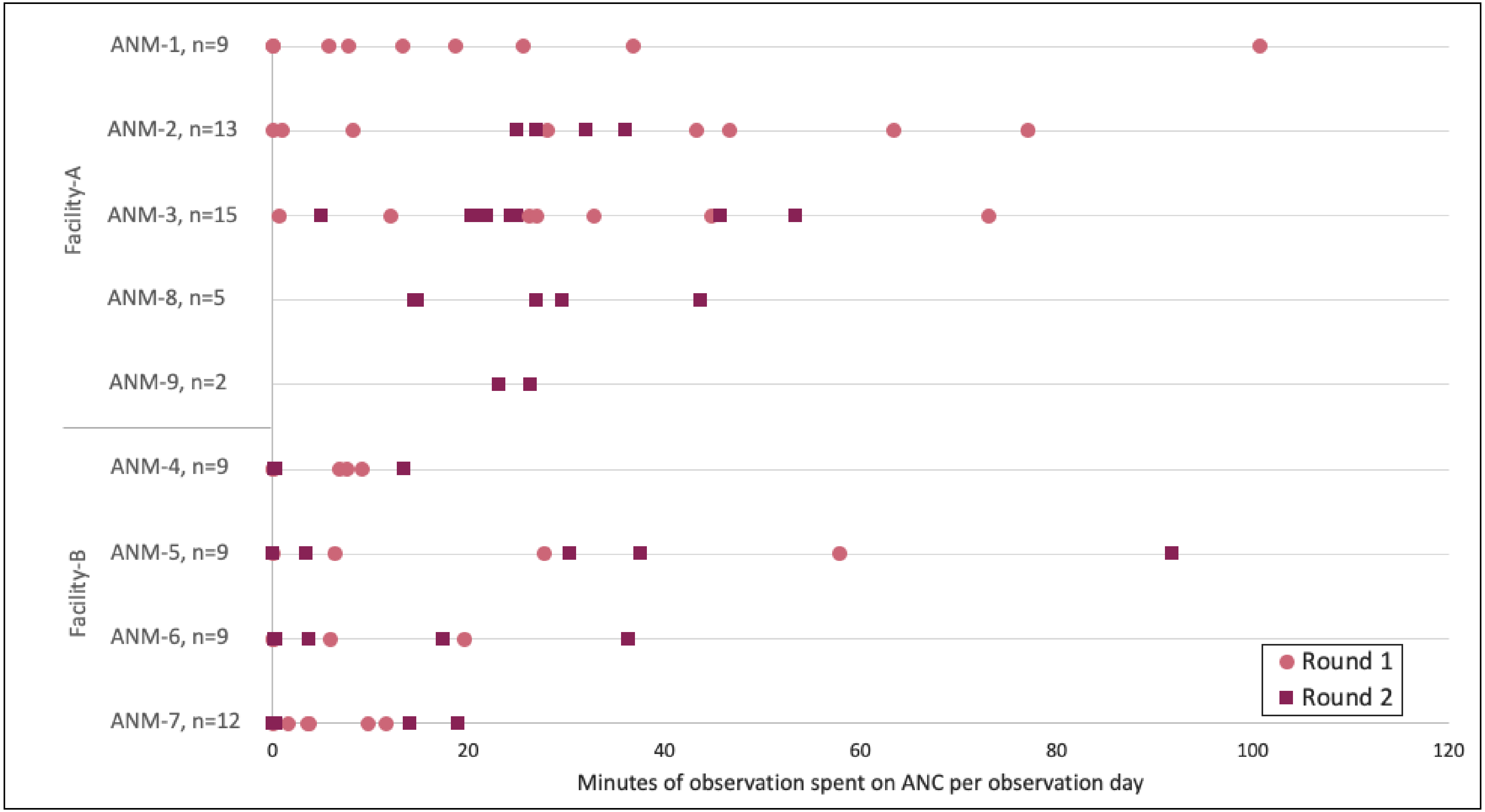

Figure S2 *Minutes spent on ANC per observation day by ANM*

Figure S3 *Start and end times during the workday of non-work activity periods greater than or equal to 10 minutes in duration for all ANMs observed at baseline (round 1)*
Panel A *- Facility-A (mIRA EDSS)*

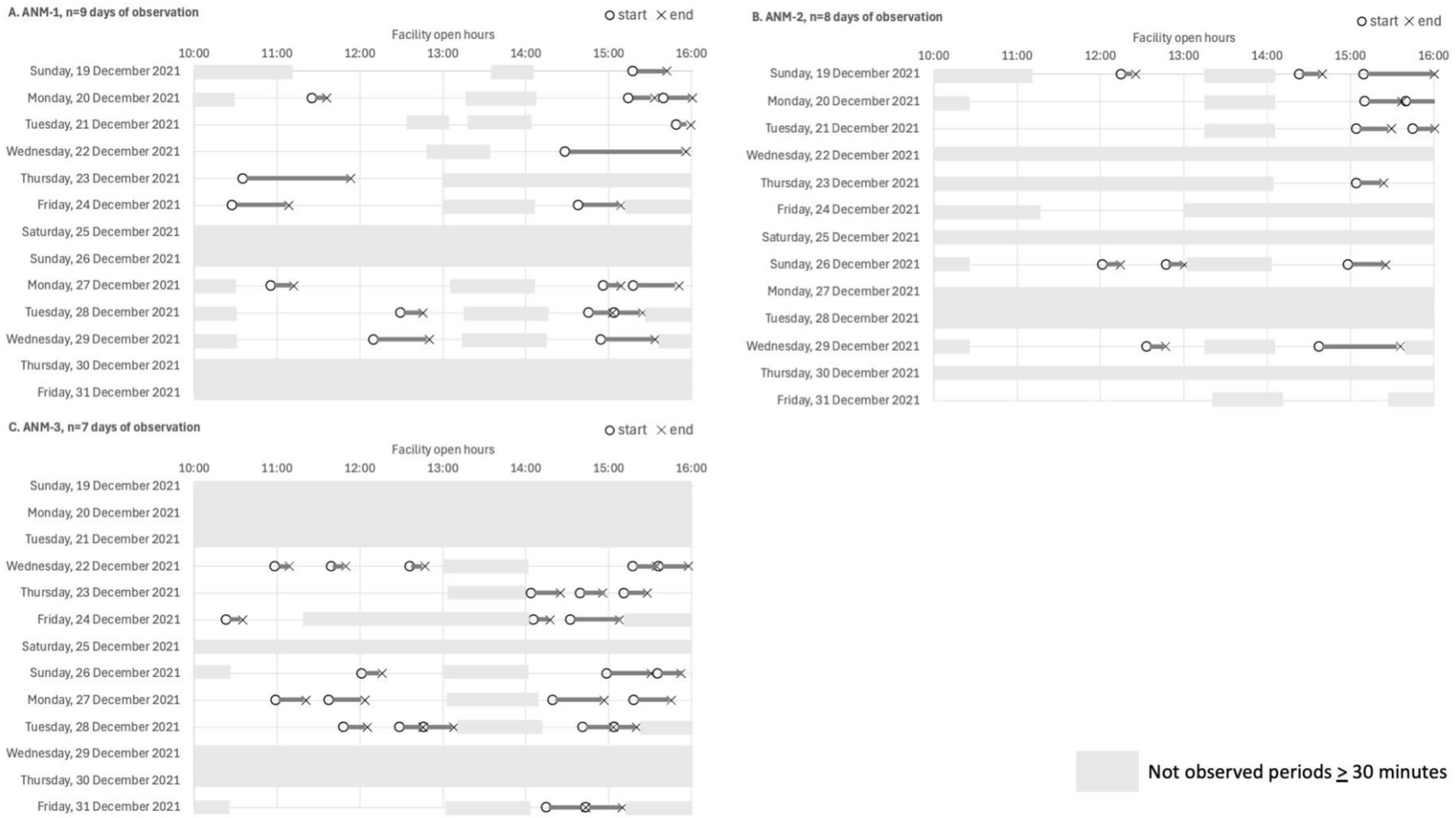

***Figure S3**, continued.* ***Panel B** – Facility-B (WHO EDSS)*

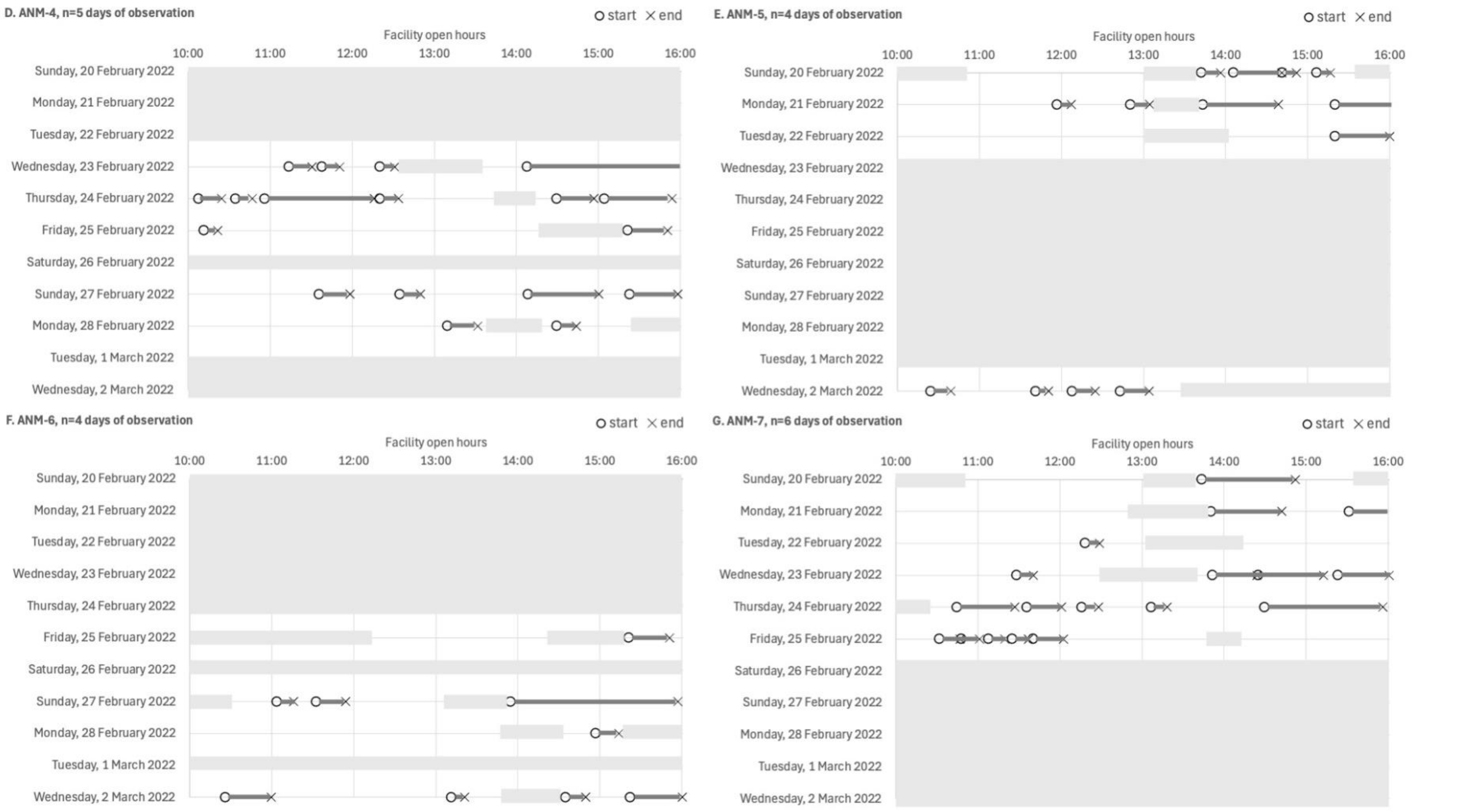

Supplement: Supplementary file 1 — Additional file 1. [file 12911_2025_2868_MOESM1_ESM.pdf]
