# Supplementary material for: Workload in antenatal care before and after implementation of an electronic decision support system: an observed time-motion study of healthcare providers in Nepal
Source: BMC Med Inform Decis Mak. 2025 Feb 18;25:87. doi: 10.1186/s12911-025-02868-1 (PMC11834578; doi:10.1186/s12911-025-02868-1)
Supplement: Supplementary file 2 — Additional file 2. [file 12911_2025_2868_MOESM2_ESM.pdf]

# Research checklist

## *STAMP (Suggested Time and Motion Procedures)*

| Area and element | Description (from Zheng et al. 2011) | Section |
|---|---|---|
| *Intervention* | | |
| Type | The system studied (intervention) | Background |
| System genre | Origin or lineage of the system (eg, commercial product, homegrown system, open source software) | Background |
| Maturity | Time elapsed since intervention, including the amount of time that study subjects have been exposed to the intervention | Discussion |
| *Empirical setting* | | |
| Institution type | Type of the healthcare facility or facilities where empirical observations are conducted (eg, academic vs non-academic) | Methods |
| Care area | Area of patient care services (eg, inpatient, outpatient, emergency department) | Background |
| Locale | Geographic characteristics (eg, urban vs rural) | Methods |
| *Research design* | | |
| Protocol | Research protocol (eg, RCT, before and after, after only) | Methods |
| Duration | Length of fieldwork (eg, whether all observations are completed within a month, or occur sporadically over the course of a year) | Results |
| Shift distribution | Clinical shifts observed (eg, morning, afternoon, night, if applicable) | Methods |
| Observation hours | Total number of direct observation hours, in addition to how the hours are distributed across study phases or RCT study arms (if applicable) | Results |
| *Task category* | | |
| Definition and classification | Definition of tasks and description of all major and minor task categories | Methods |
| Acknowledgment of prior work | Acknowledgment of task classification schemas previously used in the same or similar settings, and justifications if modifications are made | Methods |
| New development | Development and validation of task definition and task classification, if no prior work can be leveraged | n/a |
| *Observer* | | |
| Size of field team | Total number of independent human observers | Methods |
| Training | Type and amount of training provided to human observers, including pre-study pilot observation sessions | Methods |
| Background | Professional background of observers (eg, residents, nurses, industrial engineering students) and their prior experiences in conducting observational studies in clinical settings | Methods |
| Inter-observe uniformity | If and how inter-observer agreements are calibrated | Methods |
| Continuity | Continuity of observers across multiple study phases (if applicable) | Methods |

| | | |
|---|---|---|
| Assignment | How observers are assigned to shadow different research subjects and in particular, research subjects enrolled in different study phases or RCT study arms (if applicable) | n/a |
| *Subject* | | |
| Size | Number of research subjects enrolled | Results |
| Recruitment and randomization | How research subjects are recruited (and randomized, if applicable) | Methods |
| Continuity | Continuity of subjects across multiple study phases (if applicable) | Results |
| Background | Background information about research subjects such as clinician type and level of training (eg, residents vs attending physicians); if conditions allow, other individual characteristics such as gender, age, and computer literacy | Results/ Supplementary Material |
| *Data recording* | | |
| Multitasking | If and how multi-tasking is taken into account; in particular, if only the primary task is recorded or all concurrent tasks are recorded | Methods |
| Non-observed periods | If there are periods of time not covered by independent observers | Methods |
| Between task transition | If and how transition periods between consecutive tasks are handled | Methods |
| Collection tool | Device and software used to collect field data, for example, the AHRQ tool, WOMBAT, and the medical work assessment tool developed by Mache et al | Methods |
| *Data analysis* | | |
| Definition of key measures | Key measures used in analysis and results reporting, for example, average time spent on ordering activities vs on direct patient care | Methods |
| Analytical methods | Statistical or other types of analytical methods used to analyze the data | Methods |

Adapted from:
Zheng K, Guo MH, Hanauer DA. Using the time and motion method to study clinical work processes and workflow: methodological inconsistencies and a call for standardized research. J Am Med Inform Assoc JAMIA. 2011;18(5):704–10.
